# MitoQ supplementation prevent long-term impact of maternal smoking on renal development, oxidative stress and mitochondrial density in male mice offspring

**DOI:** 10.1038/s41598-018-24949-0

**Published:** 2018-04-26

**Authors:** Suporn Sukjamnong, Yik Lung Chan, Razia Zakarya, Long The Nguyen, Ayad G. Anwer, Amgad A. Zaky, Rachana Santiyanont, Brian G. Oliver, Ewa Goldys, Carol A. Pollock, Hui Chen, Sonia Saad

**Affiliations:** 1 School of Life Sciences, Faculty of Science, University of Technology Sydney, Sydney, NSW 2007 Australia; 2 Department of Clinical Chemistry, Faculty of Allied Health Sciences, Chulalongkorn University, Bangkok, Thailand; 3 Respiratory Cellular and Molecular Biology, Woolcock Institute of Medical Research, Sydney, NSW 2037 Australia; 4 Renal group Kolling Institute, Royal North Shore Hospital, St Leonards, NSW 2065 Australia; 5 ARC Centre of Excellence for Nanoscale Biophotonics, Macquarie University, North Ryde, 2109 NSW Australia

## Abstract

To investigate the effect of maternal MitoQ treatment on renal disorders caused by maternal cigarette smoke exposure (SE). We have demonstrated that maternal SE during pregnancy increases the risk of developing chronic kidney disease (CKD) in adult offspring. Mitochondrial oxidative damage contributes to the adverse effects of maternal smoking on renal disorders. MitoQ is a mitochondria-targeted antioxidant that has been shown to protect against oxidative damage-related pathologies in many diseases. Female Balb/c mice (8 weeks) were divided into Sham (exposed to air), SE (exposed to cigarette smoke) and SEMQ (exposed to cigarette smoke with MitoQ supplemented from mating) groups. Kidneys from the mothers were collected when the pups weaned and those from the offspring were collected at 13 weeks. Maternal MitoQ supplementation during gestation and lactation significantly reversed the adverse impact of maternal SE on offspring’s body weight, kidney mass and renal pathology. MitoQ administration also significantly reversed the impact of SE on the renal cellular mitochondrial density and renal total reactive oxygen species in both the mothers and their offspring in adulthood. Our results suggested that MitoQ supplementation can mitigate the adverse impact of maternal SE on offspring’s renal pathology, renal oxidative stress and mitochondrial density in mice offspring.

## Introduction

It has been increasingly recognised that maternal programming during fetal development predisposes the offspring to future disease. Maternal smoking imposes a significant adverse impact on fetal renal development that determines the future risk of chronic kidney disease (CKD) in adulthood^1^. Human studies have shown that intrauterine exposure to cigarette smoke (SE) is closely linked to impaired fetal renal growth^2^. Maternal smoking is associated with a 1.24-times increased risk of child proteinuria compared with offspring of non-smoking mothers^3^. These phenomena have also been confirmed in our mouse model of maternal smoking, which demonstrated that maternal SE leads to renal underdevelopment in offspring at birth and renal dysfunction in adulthood^4^.

Mitochondria are intracellular organelles that generate the energy required for cellular functions through oxidative phosphorylation, which involves a series of oxidation-reduction reactions. During this process, reactive oxygen species (ROS) are released as a by-product. Thus, mitochondria are the major source of ROS during energy synthesis^5^, which is subsequently cleared by the endogenous antioxidants, such as manganese superoxide dismutase (MnSOD). Mitochondrial abnormalities, such as the accumulation of mitochondrial DNA mutations and damaged mitochondria structure due to metabolic stress, can overconsume the antioxidant enzymes or impair the production of antioxidants leading to oxidative stress which in turn triggers pro-inflammatory response^6,7^. Renal tubular cells contain abundant mitochondria, therefore mitochondrial density plays a fundamental role in the pathogenesis of kidney diseases. Growing evidence suggests that mitochondrial damage is implicated in the pathophysiology of renal diseases^8,9^. It has been reported that nicotine can accumulate in the kidney^10^. Several studies indicated that maternal smoking is closely related to increased levels of oxidative stress in the mothers, infants and newborns^11,12^, and reduced levels of the antioxidant enzymes superoxide dismutase (SOD) in the arteries of offspring from nicotine treated rats^13^. Moreover, we also demonstrated that oxidative stress and mitochondrial dysfunction are closely associated with the adverse effects of maternal smoking on the kidney pathology in the male offspring^14,15^.

Coenzyme Q10 (CoQ10) is a mitochondrial endogenous antioxidant. It has been shown that CoQ10 supplementation in mice can lower hepatic oxidative stress and inflammation associated with diet-induced obesity in mice^16^. Amniotic fluid CoQ10 levels are significantly lower among women delivering preterm babies, a risk which is increased by maternal smoking^17,18^. In addition, plasma CoQ10 levels are reduced in smokers^19^. However CoQ10 is not a viable treatment option due to poor bioavailability and delayed mitochondrial uptake^20^. Mitoquinone mesylate, also known as MitoQ, is a mitochondria-targeted antioxidant. It consists of a ubiquinone moiety, the same structure to the ubiquinone found in CoQ10, which allows its rapid uptake and accumulation in the mitochondria to restore the antioxidant efficacy of the mitochondrial respiratory complex^21^. As such, it has been reported that MitoQ has a protective role against oxidative damage-related pathologies in metabolic^22^ and neurodegenerative diseases^23^. Moreover, our previous study demonstrated that maternal MitoQ supplementation during pregnancy and lactation is beneficial in reducing lung inflammatory and oxidative stress responses caused by maternal SE in the adult offspring^24^. Therefore, this study aimed to investigate whether maternal MitoQ supplementation can also mitigate the adverse impact on renal disorders caused by SE and whether the benefits of MitoQ administered to the SE mother are transmitted to the fetus and result in reduced future risk of CKD.

## Materials and Methods

### Animal experiments

The animal experiments were approved by the Animal Care and Ethics Committee at the University of Technology Sydney (ACEC#2014-638 and #2016-419). All protocols were performed according to the Australian National Health & Medical Research Council Guide for the Care and Use of Laboratory Animals. Female Balb/c mice (8 weeks) were housed at 20 ± 2 °C and maintained on a 12:12 hour light/dark cycle with ad libitum access to standard laboratory chow and water. After the acclimatisation period, mice were divided into three groups: SHAM (exposed to air), SE (exposed to cigarette smoke from 2 cigarettes twice daily, 6 weeks before mating and throughout gestation and lactation, as previously described^4^), and SEMQ (SE mothers supplied with MitoQ (500 µM in drinking water^25–27^) during gestation and lactation). Male breeders and suckling pups stayed in the home cage when the mothers were exposed to normal air or cigarette smoke. Pups were weaned at postnatal day 20 and maintained without additional intervention.

Since we have previously demonstrated that maternal SE have a greater impact on the male offspring^28^, only male offspring were assessed in this study. One cohort of pups were randomly selected at postnatal day 1 from each litter to prevent selection bias^29^. The rest of the pups were kept to week 13. The birthweight of the latter group was not measured to avoid disturbance to the new born litter and mothers and problems with attachment which may influence later results^30^. Briefly, male offspring were euthanized (4% isoflurane, 1% O_2_, Veterinary companies of Australia, Kings Park, NSW) at adulthood (13 weeks). A terminal urine collection was undertaken via direct bladder puncture and the blood was collected via cardiac puncture after mice were anesthetized. The kidney tissues were collected and stored at −80 °C for later analysis.

### Albumin and creatinine assays

The levels of urinary albumin and creatinine were measured using Murine Microalbuminuria ELISA kit (Albuwell M) and Creatinine Companion kit, respectively (Exocell Inc, PA, USA) following the manufacturer’s instructions.

### Kidney histology

Kidney structure was examined in the male offspring at 13 weeks as previously described. Briefly, fixed kidney samples were embedded in paraffin and sectioned. Kidney sections were stained with hematoxylin and eosin (H&E) and periodic acid-Schiff (PAS). Glomerular number and size were assessed as we have previously described^4^ and quantitated using Image J software (National Institute of Health, Bethesda, Maryland, USA).

### Confocal Microscopy Imaging

Confocal laser scanning microscopy images of frozen kidney sections were acquired using Leica SP2 confocal laser scanning microscope (Leica, Wetzlar, Germany). Data was generated from 5–6 animals/group. Four to 6 Images were collected from each kidney and averaged before the analysis. All imaging parameters including laser intensities, Photomultiplier tubes voltage and pinholes were kept constant during imaging. For total reactive oxygen species (ROS) detection, CellROX Deep Red (Thermo Fisher Scientific, Australia) was used at 5 µM final concentration, images were acquired at 633 nm excitation wavelength and detected in the 640–680 nm emission range. MitoTracker Green (Thermo Fisher Scientific, Australia) was used for staining the mitochondria at 200 nM final concentration, Images were acquired at 488 nm excitation wavelength and detected in the 510–550 nm emission range.

### Western blotting

Kidney tissues were homogenized in lysis buffer with phosphatase inhibitors (Thermo Fisher Scientific, CA, USA). Protein concentrations were measured using DC Protein assay (Bio-rad, Hercules, CA, USA). Equal amount of proteins (20 μg) were separated on 4–12% Criterion™ XT Bis-Tris Protein Gel (Bio-rad, Hercules, CA, USA) and transferred to PVDF membranes. The membranes were blocked with TBS-0.05% Tween 20 (TBS-T) containing 5% BSA or skim milk for 1 h, before incubation with primary antibodies against endogenous antioxidant Manganese superoxide dismutase (MnSOD, 1:2000, Millipore, Billerica, MA, USA), translocase of the outer membrane-20 (TOM-20, 1:2000, Santa Cruz Biotechnology), fibronectin (1:1000, Abcam, Cambridge, UK), phospho-extracellular signal-regulated kinase-1/2 (Erk1/2, 1:1000, Cell Signaling Technology Inc), Erk1/2 (1:1000, Cell Signaling Technology Inc), phospho-JNK (1:1000, Cell Signaling Technology Inc, MA, USA), c-JUN N-terminal kinase (JNK, 1:500, Cell Signaling Technology Inc), phospho-p38 Mitogen-activated protein kinase (MAPK, 1:1000, Cell Signaling Technology Inc), p38 MAPK (1:1000, Cell Signaling Technology Inc), transcription factor nuclear factor-κ-light-chain-enhancer of activated B cells (NFκB, 1:1000, Cell Signaling Technology Inc), phospho-NFκB (1:1000, Cell Signaling Technology Inc), F4/80 (1:1000, Sigma Aldrich, New South Wales, Australia), Collagen I (1:500, Santa Cruz Biotechnology, Texas, USA), Collagen III (1:500, Santa Cruz Biotechnology, Texas, USA) and Collagen IV (1:500, Santa Cruz Biotechnology, Texas, USA) overnight at 4 °C, then followed by secondary antibodies (peroxidase-conjugated goat anti-mouse or anti-rabbit IgG or rabbit anti-goat IgG,1:2000,Santa Cruz Biotechnology Inc). The blots were then incubated in Super Signal West Pico Chemiluminescent substrate (Thermo Fisher Scientific, CA, USA) and the membranes were then visualized by an Amersham Imager 600 (GE Healthcare, NSW, Australia). Protein band density determined using ImageJ software (National Institute of Health, Maryland, USA) was used for densitometry, and β-actin (1:5000, Santa Cruz Biotechnology, Texas, USA) was used as the control.

### Quantitative real-time PCR

Total mRNA was isolated from kidney tissues using TRIzol Reagent (LifeTechnologies, CA, USA). First strand cDNA was generated using M-MLV Reverse Transcriptase, RNase H, Point Mutant Kit (Promega, Madison, WI, USA). Genes of interest were measured using pre-optimized SYBR green primers (Sigma-Aldrich) and RT-PCR master mix (LifeTechnologies, CA, USA). The primers used in real-time RT-PCR experiments were as follows: macrophage chemoattractant protein (MCP)-1 forward primer: 5′-GTTGTTCACAGTTGCTGCCT-3′, and reverse primer: 5′-CTCTGTCATACTGGTCACTTCTAC-3′. Interleukin (IL)-1α, IL-6 and cluster of differentiation (CD) 68 mRNA expressions were measured using Taqman probe (IL-1α: ACCTGCAACAGGAAGTAAAATTTGA, NCBI gene references: NM_010554.4, mCT192405.0, BC003727.1, ID: Mm00439620_m1; IL-6: ATGAGAAAAGAGTTGTGCAATGGCA, NCBI gene references: NM_031168.1, X06203.1, X54542.1, ID: Mm00446190_m1; CD68: CACTTCGGGCCATGTTTCTCTTGCA, NCBI gene references: NM_001291058.1, ID: Mm03047343_m1). The average expression of the control group was assigned as the calibrator against which all other samples were expressed as fold difference. The 18S rRNA was used as the house-keeping gene for all gene of interest.

### Mitochondrial DNA copy number

Genomic DNA was extracted from renal tissue using the DNeasy blood and tissue kit (Qiagen). The content of mtDNA was calculated using real-time quantitative PCR by measuring the threshold cycle ratio (∆Ct) of the mitochondrial-encoded gene cytochrome c oxidase subunit 1 (COX1) (forward primers 5′-ACTATACTACTACTAA-CAGACCG-3′, reverse primers 5′-GGTTCTTTTTTTCCGGAGTA-3′) vs. the nuclear-encoded gene cyclophilin A (forward primers 5′-ACACGCCATAATGGCACTGG-3′, reverse primers 5′-CAGTCTTGGCAGTGCAGAT-3′ as we have previously shown^15^.

### ATP assay

ATP determination kit (Thermo Fisher Scientific, CA, USA) was used to extract ATP according to manufacturer instructions. In brief, kidney tissues (15–20 mg) were homogenized in 0.5 ml ice-cold Phenol-TE (Sigma-Aldrich, New South Wales, Australia). Chloroform (200 μl) and de-ionised water (200 μl) were added and followed by twenty seconds shaking. Aqueous phase was extracted and ATP was determined with luciferin-luciferase assay.

### Statistical analysis

Results are presented as the mean ± S.E.M. The differences between the groups were analysed by one-way ANOVA followed by post hoc Bonferroni test (Prism 7, Graphpad CA, USA). The differences were considered statistically significant at P < 0.05.

## Results

### Effects of cigarette smoke exposure on the mothers

**Figure 1:**
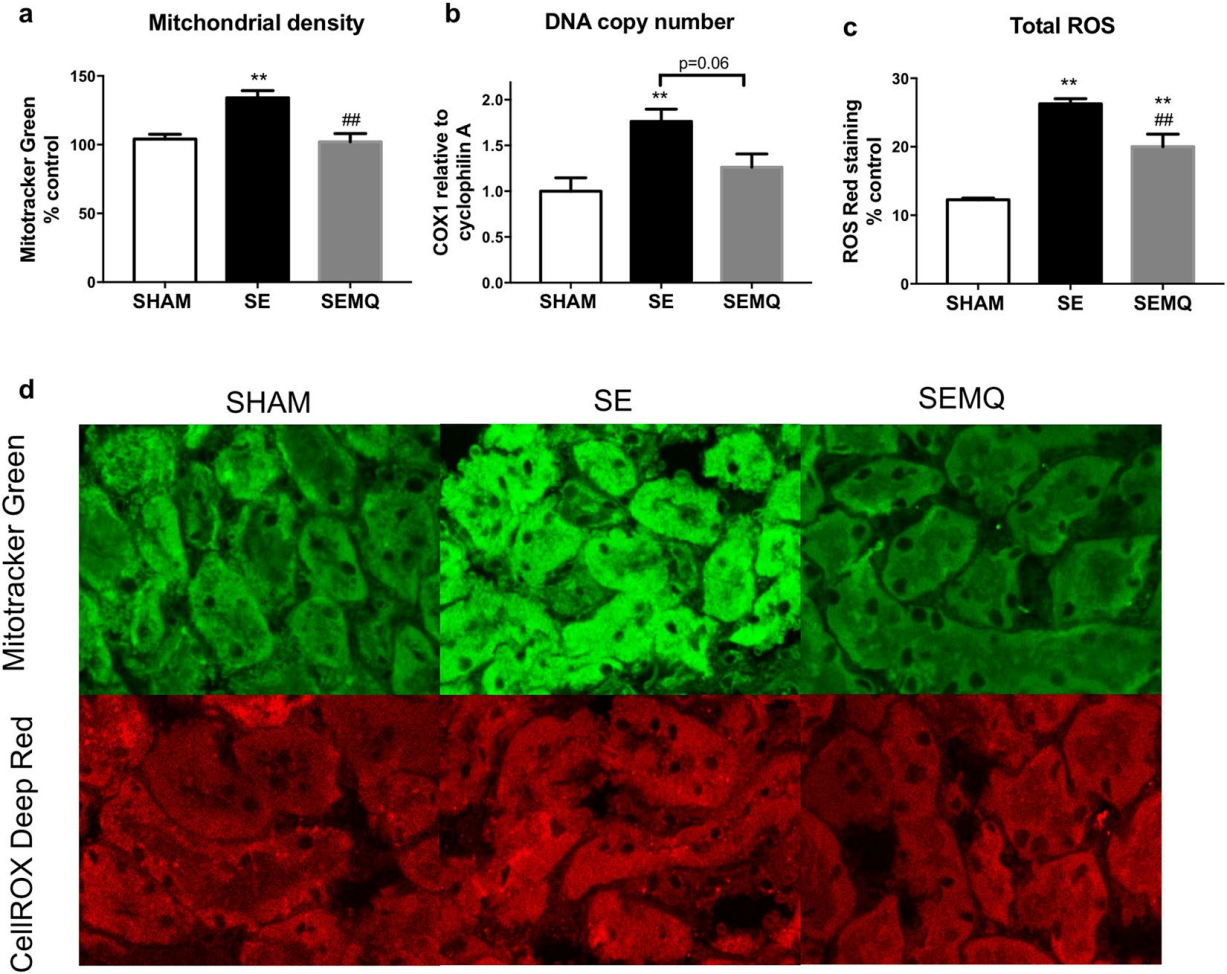
Renal mitochondrial density (**a**), mitochondrial DNA copy number (**b**) and total ROS level (**c**) in the mothers. Results are expressed as mean ± SE.**P < 0.01 SE vs Sham, ^##^*P* < 0.01 SEMQ vs SE. SE: cigarette smoke exposure; SEMQ: cigarette smoke exposure with MitoQ supplementation. Representative confocal images of (**a** and **c**) showing Mitotracker and Cell Rox staining in the SHAM, SE and SEMQ groups respectively (**d**).

Results in Table 1 show that body weight was not different between the SE mothers and control mothers. Kidney mass was marginally reduced without statistical significance in the SE mothers. Mitochondrial density, total ROS levels, and mitochondrial copy number were significantly increased in the kidney’s from the SE mothers (P < 0.01 vs SHAM, Fig. 1).

**Table 1:** Body weight and kidney weights of the mothers and offspring at postnatal day 1 and 13 weeks.

Mother	Control (n = 9)	SE (n = 10)	SEMQ (n = 9)
Body weight (g)	21.8 ± 0.7	21.1 ± 0.9	21.2 ± 0.3
Kidney weight (g)	0.166 ± 0.006	0.148 ± 0.006	0.157 ± 0.003
Kidney % body weight	0.77 ± 0.04	0.71 ± 0.03	0.74 ± 0.02
**Offspring P1**	**(n = 14)**	**(n = 11)**	**(n = 7)**
Body weight (g)	1.51 ± 0.03	1.30 ± 0.06**	1.65 ± 0.05^##^
Kidney weight (g)	0.009 ± 0.0005	0.007 ± 0.0004*	0.009 ± 0.0006^#^
Kidney % body weight	0.57 ± 0.03	0.55 ± 0.04	0.55 ± 0.03
**Offspring 13 weeks**	**(n = 19)**	**(n = 20)**	**(n = 14)**
Body weight (g)	25.2 ± 0.2	24.2 ± 0.2 **	25.1 ± 0.2^##^
Kidney weight (g)	0.195 ± 0.004	0.186 ± 0.003	0.198 ± 0.004
Kidney % body weight	0.77 ± 0.02	0.77 ± 0.02	0.79 ± 0.02

Results are expressed as mean ± SE. **P < 0.01 SE vs Sham, ^##^*P* < 0.01 SEMQ vs SE.

MitoQ supplementation during gestation and lactation significantly reversed the impact of SE on mitochondrial density (P < 0.01 vs SE, Fig. 1a). In addition, renal DNA copy number in the SEMQ mothers was similar as the SHAM mothers (Fig. 1b). Maternal MitoQ administration also significantly ameliorated ROS level in the kidneys (P < 0.01 SEMQ vs SE, Fig. 1c).

### Effect of maternal cigarette smoke exposure on the growth of the male offspring

At postnatal day 1, body weight and kidney mass were significantly reduced in the male offspring from the SE mothers (P < 0.01 and P < 0.05 vs SHAM, respectively; Table 1). A lower body weight was maintained until 13 weeks of age (P < 0.01, Table 1) but kidney mass was only marginally reduced without statistical significance in offspring of SE mothers. This is consistent with our previous study using the same model^4^.

Maternal MitoQ supplementation significantly enhanced body weight at P1 and normalised the body weight at week13 of the SEMQ offspring (P < 0.01, Table 1). Moreover, kidney mass was significantly normalised in the SEMQ offspring at P1 (P < 0.05 vs SE, Table 1). Interestingly, there were fewer male offspring in the SEMQ group in comparison to both SE and control groups.

### Effect of maternal cigarette smoke exposure on kidney development

**Figure 2:**
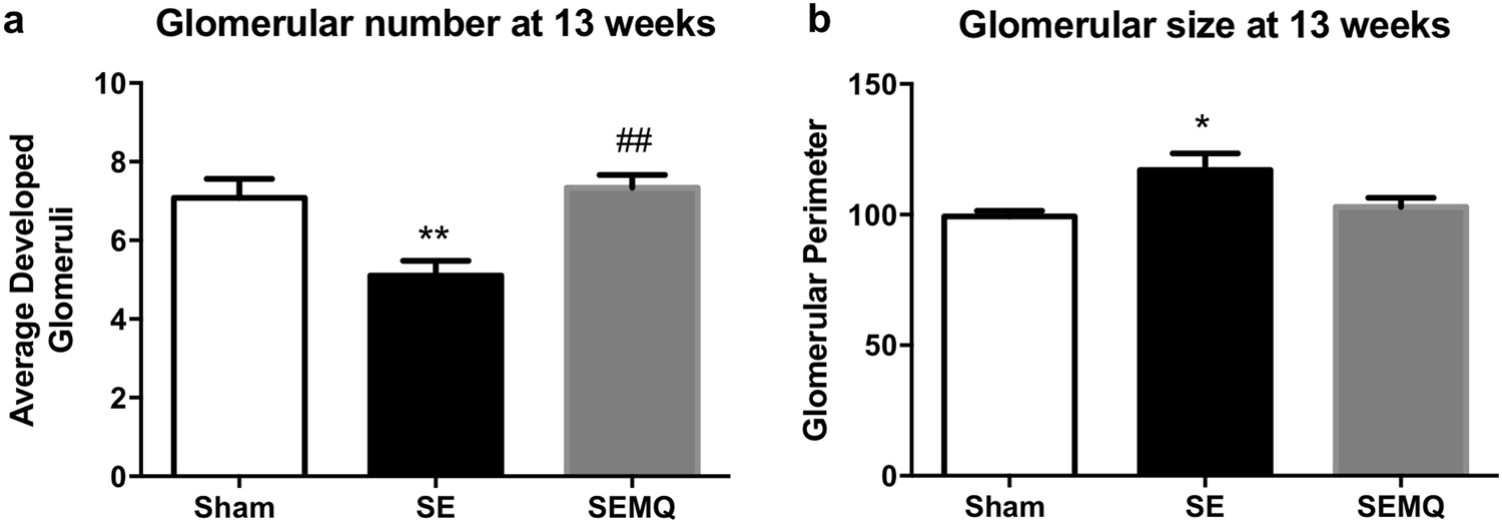
Glomerular number and size in the male offspring at 13 weeks. Results are expressed as mean ± SE. *P < 0.05, **P < 0.01 SE vs Sham, ^##^*P* < 0.01 SEMQ vs SE. SE: cigarette smoke exposure; SEMQ: cigarette smoke exposure with MitoQ supplementation.

At 13 weeks, the average number of glomeruli was significantly decreased in the SE offspring compared to the SHAM offspring (P < 0.01, Fig. 2a). The mature glomerular size in the SE offspring was also significantly larger than those of the offspring from the SHAM mothers (P < 0.05, Fig. 2b). Maternal MitoQ supplementation normalised glomerular number (P < 0.01 vs SE) and size in the SEMQ offspring (Fig. 2a,b).

### Effect on renal inflammatory and fibrotic markers and kidney function

**Figure 3:**
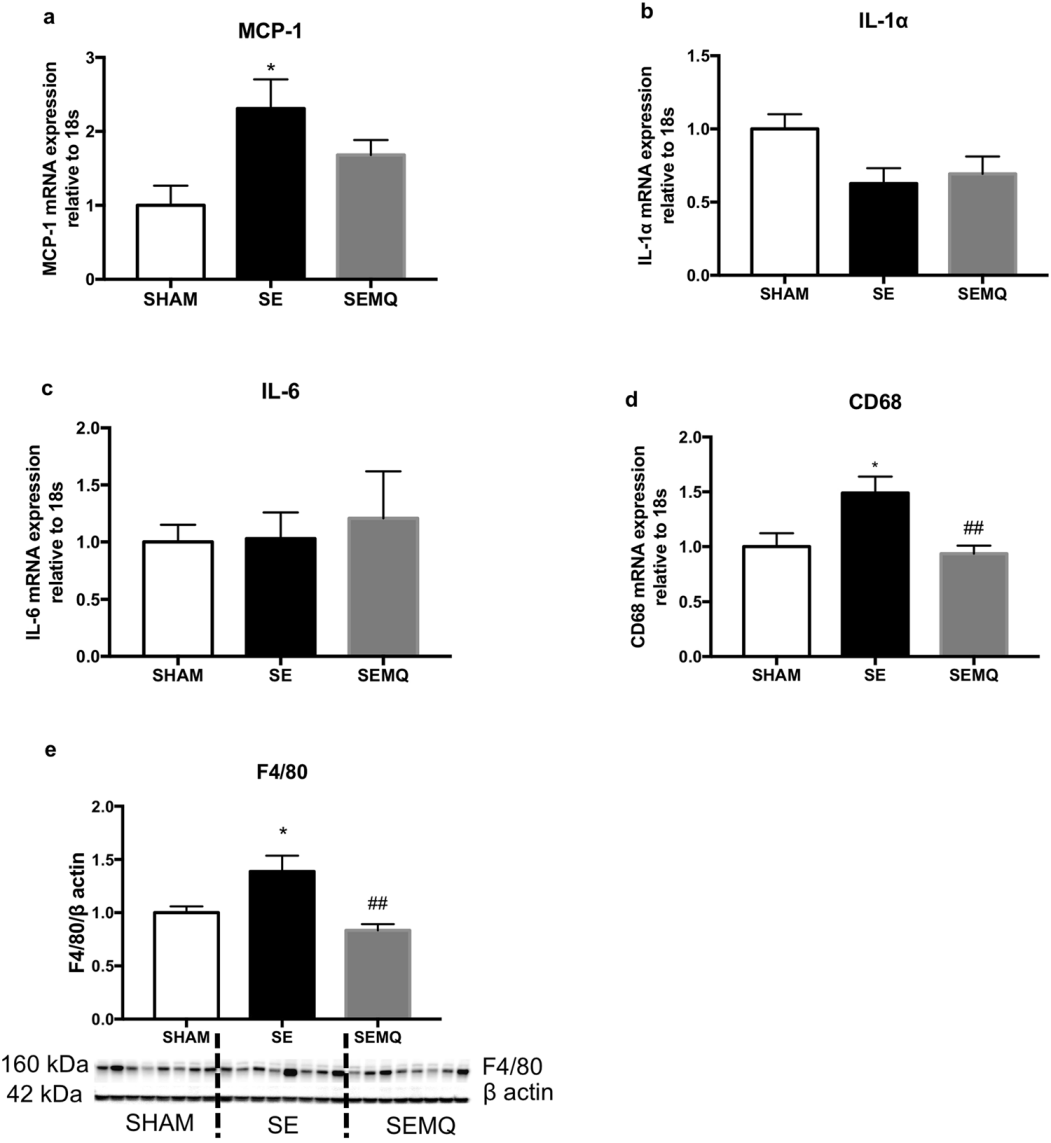
Renal MCP-1 mRNA expression (**a**), IL-1α mRNA expression (**b**), IL-6 mRNA expression (**c**), CD68 mRNA expression (**d**), F4/80 protein levels (**e**) in the offspring at 13 weeks. Whole gel images (**e**) in Supplementary Fig. 3. Results are expressed as mean ± SE. *P < 0.05, SE vs Sham. ^##^*P* < 0.01; ^#^P < 0.05, SEMQ vs SE. SE: cigarette smoke exposure; SEMQ: cigarette smoke exposure with MitoQ supplementation.

**Figure 4:**
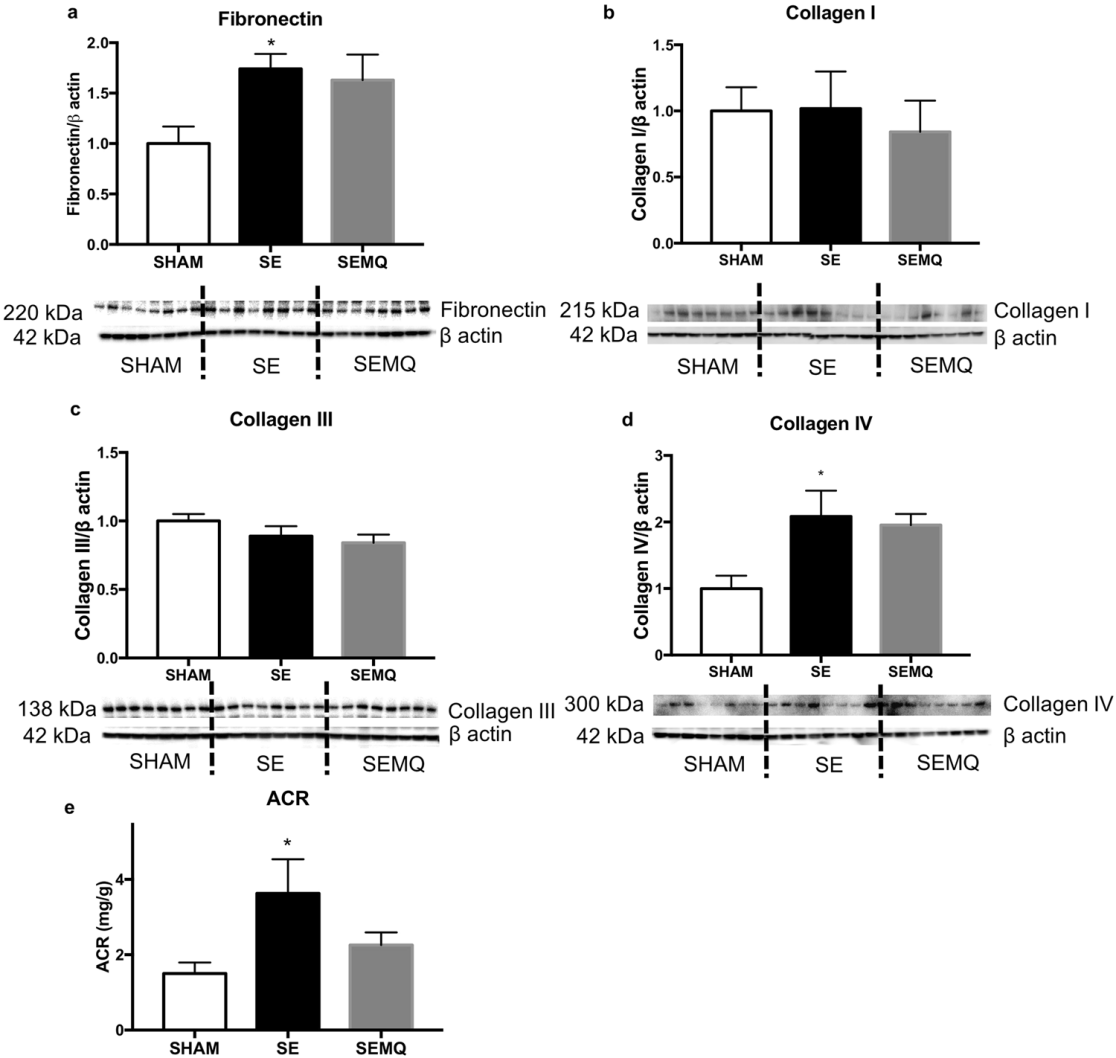
Renal fibronectin protein levels (**a**), collagen I protein levels (**b**), collagen III protein levels (**c**), collagen IV protein levels (**d**), and Urinary Albumin to creatinine ratio (ACR) in the offspring (**e**) at 13 weeks. Whole gel images (**a**–**d**) in Supplementary Fig. 4, (**a**,**b**). Results are expressed as mean ± SE. *P < 0.05, SE vs Sham. SE: cigarette smoke exposure; SEMQ: cigarette smoke exposure with MitoQ supplementation.

Renal mRNA expression of the pro-inflammatory markers MCP-1 and CD68, in addition to the protein levels of F4/80, mice macrophage marker were significantly increased in the offspring kidneys due to maternal SE (P < 0.05 vs SHAM offspring, Fig. 3a,d,e). The levels of the pro-fibrotic marker fibronectin and collagen IV protein were also significantly increased in the offspring kidneys due to maternal SE (P < 0.05 vs SHAM offspring, Fig. 4a,d). IL-1α and IL-6 expression, as well as Collagen I,III protein levels were not changed by maternal SE (Figs 3b,c, 4b,c). Maternal MitoQ administration ameliorated MCP-1 expression although this did not reach statistical significance (Fig. 3a); whereas CD68 expression and F4/80 protein level were normalised in the SEMQ offspring (P < 0.01 vs SE, Fig. 3d; P < 0.05 vs SE, Fig. 3e).

The urinary albumin-to-creatinine ratio as a marker of renal damage was significantly higher in the SE group (P < 0.05, Fig. 4e). Maternal MitoQ supplementation showed a trend to normalization of urinary albumin-to-creatinine ratio. However, this was not significant (Fig. 4e).

### Effect on renal mitochondrial and stress markers in the offspring

**Figure 5:**
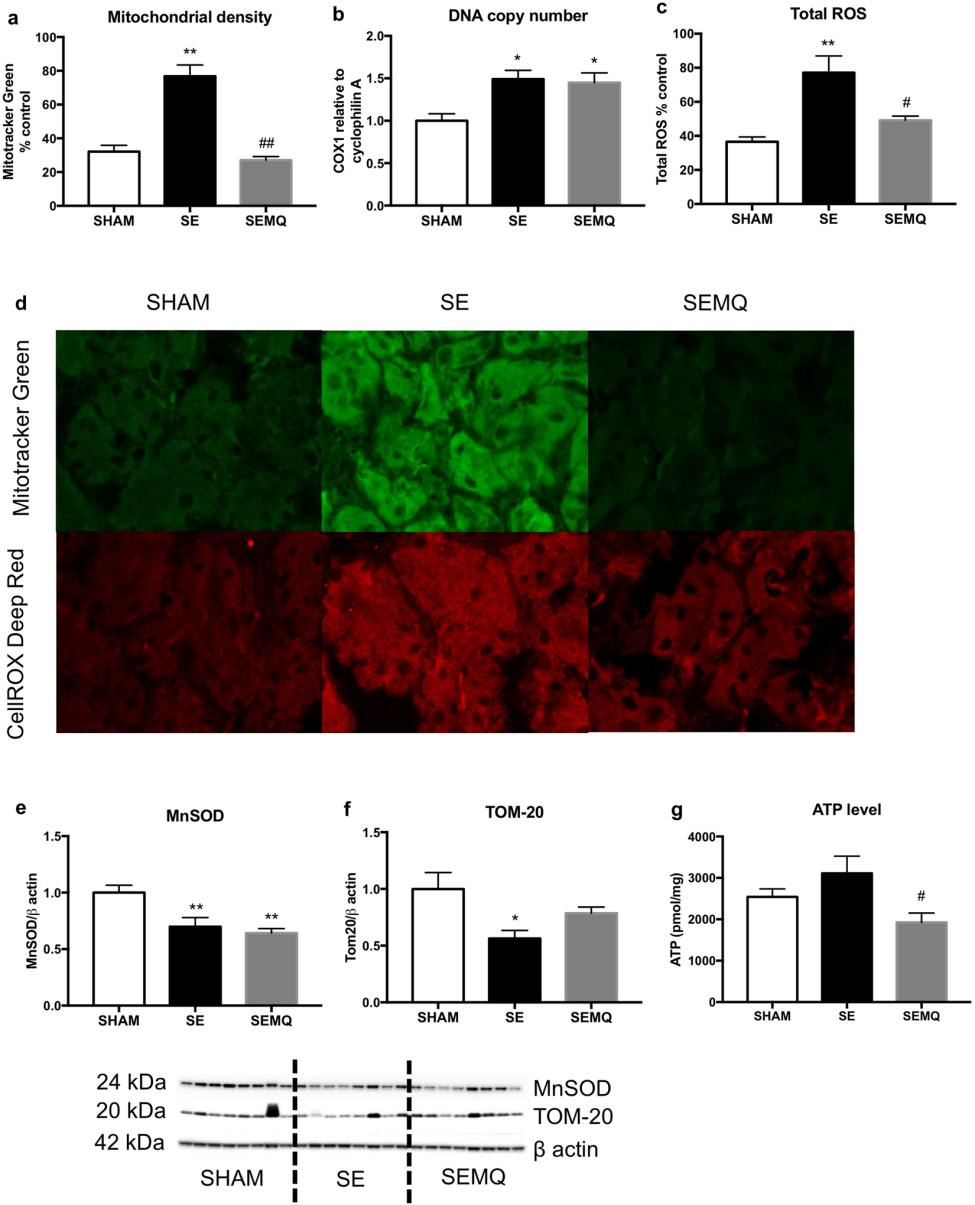
Renal cellular mitochondrial density (**a**), mitochondrial DNA copy number (**b**), total ROS (**c**), Representative confocal images of (**a** and **c**) showing Mitotracker and Cell Rox staining in the SHAM, SE and SEMQ groups respectively (**d**), mitochondrial MnSOD (**e**), and TOM-20 (**f**), and ATP (**g**) in the male offspring at 13 weeks. Whole gel images of (**e**,**f**) in Supplementary Fig. 5. Results are expressed as mean ± SE. *P < 0.05, **P < 0.01 SE vs Sham, ^#^P < 0.05, ^##^*P* < 0.01 SEMQ vs SE. SE: cigarette smoke exposure; SEMQ: cigarette smoke exposure with MitoQ supplementation.

Altered mitochondrial number and DNA content have been proposed as a surrogate of mitochondrial function. Here, mitochondrial density and DNA copy number were significantly increased in the offspring from the SE mothers (P < 0.01 and P < 0.05 vs SHAM offspring, respectively; Fig. 5a,b). As such, total ROS level was significantly increased in SE offspring’s kidneys (P < 0.01 vs SE offspring, Fig. 5c), with reduced endogenous antioxidant MnSOD level (P < 0.01 vs SHAM offspring, Fig. 5e). We also investigated TOM-20, a mitochondrial outer membrane receptor for the translocation of cytosolically synthesized mitochondrial pre-proteins. Renal TOM-20 protein level was significantly reduced in the SE offspring (P < 0.05 vs SHAM offspring, Fig. 5f).

Cellular oxidative stress level was significantly reduced by maternal MitoQ treatment. Maternal MitoQ treatment normalised cellular mitochondrial density (P < 0.01 vs SE offspring) and total ROS level (P < 0.05 vs SE offspring, Fig. 5a,c) although no change in copy number was seen. It also marginally improved TOM-20 level (Fig. 5f). There was a trend of increased ATP levels in the SE offspring’s kidneys (P = 0.27 vs SHAM), which was significantly reduced by maternal MitoQ supplementation (P < 0.05 SEMQ vs SE, Fig. 5g).

### Effect on Receptors for Advanced Glycation End-products (RAGE) pathway

RAGE is a multi-ligand receptor of the immunoglobulin superfamily, which plays a role in cigarette smoke-related disease through the AGEs-RAGE axis^31,32^. Our data demonstrated that maternal SE has no effect on RAGE, p38 MAPK, ERK1/2, JNK, and NFκB in the offspring’s kidneys at week 13. MitoQ administration also has no effect on these markers (Supplementary Fig. 1).

## Discussion

Maternal smoking during pregnancy affects fetal renal development which is linked to an increased risk of CKD in the offspring in the adulthood. We have previously demonstrated that maternal SE significantly reduced renal development in the male offspring and induced renal pathology in adulthood associated with increased oxidative stress and mitochondrial dysregulation^4,14^. Such effects were male specific^28^. However, it is not clear whether this is due to the direct effect of cigarette smoke on maternal mitochondrial DNA, which can be transmitted to the offspring.

In this study, we demonstrated that SE increased mitochondrial density and maternal renal DNA copy number and as a consequence increased total ROS levels in the mothers’ kidneys. We additionally demonstrated that the administration of the mitochondrial-targeted antioxidant MitoQ during gestation and lactation can significantly reverse the impact of SE on the abovementioned renal changes. Furthermore, we demonstrated that maternal SE induced renal underdevelopment and renal dysfunction in the male offspring at adulthood associated with increased renal inflammatory markers, mitochondrial alteration and oxidative stress, which were also ameliorated by maternal MitoQ supplementation. Interestingly, mitochondrial DNA copy number and density were increased in both SE mothers and their offspring suggesting that smoking during pregnancy can alter mitochondrial DNA predisposing the offspring to future kidney disease through foetal programing.

Maternal MitoQ administration reversed the effect of maternal SE on the offspring body weight, kidney size at birth, renal development, as well as renal function in the adult offspring. Interestingly, although maternal MitoQ administration was able to reverse the effect of maternal SE on renal mitochondrial density and total ROS levels in the offspring, it had no effect on mitochondrial DNA copy number. This finding suggests that the effect of SE on mitochondrial DNA copy number in the mothers may be transmitted to the offspring as mitochondrial DNA is inherited from the maternal lineage. Such change can’t be reversed by gestational MitoQ supplementation. However, whether such effect occurs prior to gestation requires further validation by examining the females before mating, which is beyond the scope of this study. It is important to note that there were less male offspring in the SEMQ group in comparison to both SE and control groups. The reason for that is to date unclear and is worth further investigation.

Several studies have indicated that maternal cigarette smoking during pregnancy was the most common cause of fetal growth restriction and reduced size of the fetal organs^33,34^. We have previously shown, using the same animal model, that maternal SE is linked to smaller glomerular size and delayed kidney development in the male offspring^4,14^. In addition, oxidative stress and mitochondrial dysfunction are closely associated with the adverse effects of maternal smoking on offspring’s kidney pathology^14,15^. Such phenotype has also been presented in the SE offspring in this study, reflected by smaller glomerular number with adaptive enlargement of glomerular size and impaired renal function. Mitochondrial DNA can only be inherited from the mothers, not the fathers^35^. Indeed in this study, the changes in renal mitochondrial DNA copy number and density in the SE offspring mirrored that in the SE mothers. While mitochondrial number and DNA copy number were deregulated by maternal SE, renal total ROS were increased in such offspring in line with increased mitochondrial activity of ATP production, suggesting oxidative stress, which is consistent with our previous studies^14,15^. Correlatively, the level of mitochondrial endogenous antioxidant MnSOD was reduced in the offspring’s kidney in response to increased oxidative stress, with lower expression of the mitochondrial import receptor subunit (TOM-20) which may be induced by increased work load for ATP synthesis.

Oxidative stress is often linked to inflammatory responses and fibrotic changes, which were also observed in the SE offspring with increased levels of inflammatory (MCP-1, CD68 and F4/80) and fibrotic markers (fibronectin and collagen IV). The AGEs-RAGE interaction has also been shown to associate with enhanced production of intracellular ROS, which can mediate further inflammatory response^36,37^. Several studies have suggested that RAGE can also influence the pathogenesis of renal disorders^38,39^. Our previous study demonstrated that maternal SE can increase RAGE and its signalling elements, as well as promoting oxidative stress and inflammatory responses in offspring’s lung^24^. However in this study, none of the RAGE signaling elements including RAGE, p38 MAPK, ERK1/2, JNK, and NFκB, were changed in the SE offspring’s kidney. These findings suggest that the RAGE pathway does not seem to be involved in maternal SE induced inflammatory response in the offspring’s kidney. There is also evidence suggesting that inflammatory cell infiltration correlates with both the extent of renal fibrosis and the severity of renal damage in CKD^40^. Irreversible renal fibrosis is a common consequence after renal injury and leads to a gradual loss of kidney function, which is a hallmark of CKD. In this study, there was a significant increase in fibronectin level in the SE offspring’s kidney. We have previously demonstrated that maternal SE induced subtle pathological changes in the offspring’s kidneys at 13 weeks. Increased risk of CKD may prevail if the offspring are exposed to additional insult after weaning, such as obesity or diabetes. Such hypothesis requires validation in future studies.

Mitochondrial dysfunction occurs in several human disorders, which is considered as the major driver for cellular and organ failure. Adverse effects of cigarette smoke have been attributed to increased oxidative stress together with mitochondrial dysregulation, which play a key role in the progression of renal injury and development of CKD^41–44^. Therefore, therapeutic application of mitochondrial-targeted therapies may offer potential alternatives for the prevention and treatment of such conditions, instead of the generic antioxidants which are normally poorly taken up by the mitochondria. The most widely investigated mitochondria-specific antioxidant to date is MitoQ^21,45^. The beneficial effects of MitoQ have been reported in various disorders, such as metabolic disease^22^, neurodegenerative diseases^23^, kidney damage related to diabetes^46^, Parkinson’s disease^47^, and liver inflammation in hepatitis C virus infection^48^. Importantly, our previous study using the same cohort of mice demonstrated that maternal MitoQ supplementation during pregnancy and lactation is beneficial in reducing lung inflammatory and oxidative stress responses in the adult offspring caused by maternal SE^24^. As we have demonstrated that oxidative stress plays an important role in maternal SE related renal disorders in the male offspring^4,14,15^, this study extended the investigation of the impact of maternal MitoQ supplementation on renal disorders.

In the current study, our results showed that MitoQ supplementation during pregnancy can significantly mitigate small body weight due to in-utero SE. Moreover, we demonstrated that MitoQ treatment can restore smaller kidney size and glomerular numbers with nearly normalised renal function in adult offspring from the SE mother. These results suggested that MitoQ exert beneficial effects on offspring’s health, despite continuing maternal SE during gestation and lactation. Our results are consistent with earlier reports that showed that MitoQ treatment prevented renal disorders in a mouse model of type 1 diabetes^46^. Mukhopadhyay and colleagues also found that MitoQ treatment prevented renal dysfunction caused by cisplatin nephrotoxicity^49^. Such improvement in the offspring is closely related to reduced renal ROS level and normalised mitochondrial density in both mothers and offspring. Interestingly, renal MnSOD level was not increased in the offspring as a consequence of maternal administration of MitoQ. This was different to that observed in the lungs^24^, suggesting that ROS was suppressed by other antioxidative mechanisms or due to reduced mitochondrial ATP production. The impact of maternal SE on TOM-20, the mitochondrial outer membrane receptor for the translocation of cytosolically synthesized mitochondrial preproteins, was partially reversed in SE offspring, suggesting some improvement in mitochondrial function. However, mitochondrial DNA copy number was not reversed in the SEMQ offspring compared with the SEMQ mothers, suggesting oxidative stress may not be the only factor to damage mitochondrial DNA in the offspring. Foetal kidneys are likely to be more vulnerable to the damage from the chemicals in the cigarette smoke, since nicotine level is 15% higher in the foetal blood than the maternal blood^50^. This may also affect the fibronectin level in the offspring’s kidney^51^, which was also unaffected by maternal MitoQ supplementation although the inflammatory markers MCP-1, CD68, F4/80 and ROS level were reduced. As the aim of the study was to determine whether MitoQ protects the offspring from maternal smoking we did not include a sham group treated with MitoQ. Hence we are unable to be definitive about whether MitoQ may cause changes in the parameters studied in “normal’ animals. However, Rodriguez-Cuenca *et al*., have previously examined the long-term consequences of MitoQ on wild-type mice in the absence of injury and demonstrated that MitoQ has no effect on mitochondrial function, mitochondrial DNA, food consumption or whole body metabolism^25^.

In summary, our study demonstrates the beneficial effects of maternal MitoQ supplementation during gestation and lactation on renal under development and pathology by maternal SE. It also reduced renal ROS accumulation and mitochondrial density in both mothers’ and offspring’s kidneys. Although MitoQ was unable to reverse the increase in fibrotic markers, it may still protect the offspring against maternal SE induced renal pathology and potentially future CKD through reduction of inflammation and oxidative stress. This is yet to be confirmed in future human clinical trials.

## Electronic supplementary material

Supplementary information
