# Supplementary material for: MitoQ supplementation prevent long-term impact of maternal smoking on renal development, oxidative stress and mitochondrial density in male mice offspring
Source: Sci Rep. 2018 Apr 26;8:6631. doi: 10.1038/s41598-018-24949-0 (PMC5919980; doi:10.1038/s41598-018-24949-0)
Supplement: Supplementary file 1 — Supplementary information [file 41598_2018_24949_MOESM1_ESM.doc]

**Supplementary information**

**MitoQ supplementation prevent long-term impact of maternal smoking on renal development, oxidative stress and mitochondrial density in male mice offspring**

Suporn Sukjamnong^#1,2^, Yik Lung Chan^#1,3^, Razia Zakarya^1,3^, Long The Nguyen^4^, Ayad G. Anwer^5^, Amgad A.Zaky^4^, Rachana Santiyanont^2^, Brian G Oliver^1,3^, Ewa Goldys^5^, Carol A Pollock^4^, Hui Chen^1^, Sonia Saad*^4,1^.

^#^ Suporn Sukjamnong and Yik Lung Chan contributed equally to this work

1. School of Life Sciences, Faculty of Science, University of Technology Sydney, Sydney, NSW, 2007, Australia

2.Department of Clinical Chemistry, Faculty of Allied Health Sciences, Chulalongkorn University, Bangkok, Thailand

3. Respiratory Cellular and Molecular Biology, Woolcock Institute of Medical Research, Sydney, NSW, 2037, Australia

4. Renal group Kolling Institute, Royal North Shore Hospital, St Leonards, NSW 2065, Australia

5. ARC Centre of Excellence for Nanoscale Biophotonics, Macquarie University, North Ryde 2109, NSW Australia

***Corresponding author**

Dr Sonia Saad

Renal group Kolling Institute, Royal North Shore Hospital, St Leonards, NSW 2065, Australia Tel +61 2 9926 4782, Fax +61 2 9926 5715, Email: [sonia.saad@sydney.edu.au](mailto:sonia.saad@sydney.edu.au)

Keywords : Maternal smoking, renal health, oxidative stress, mitochondrial dysfunction


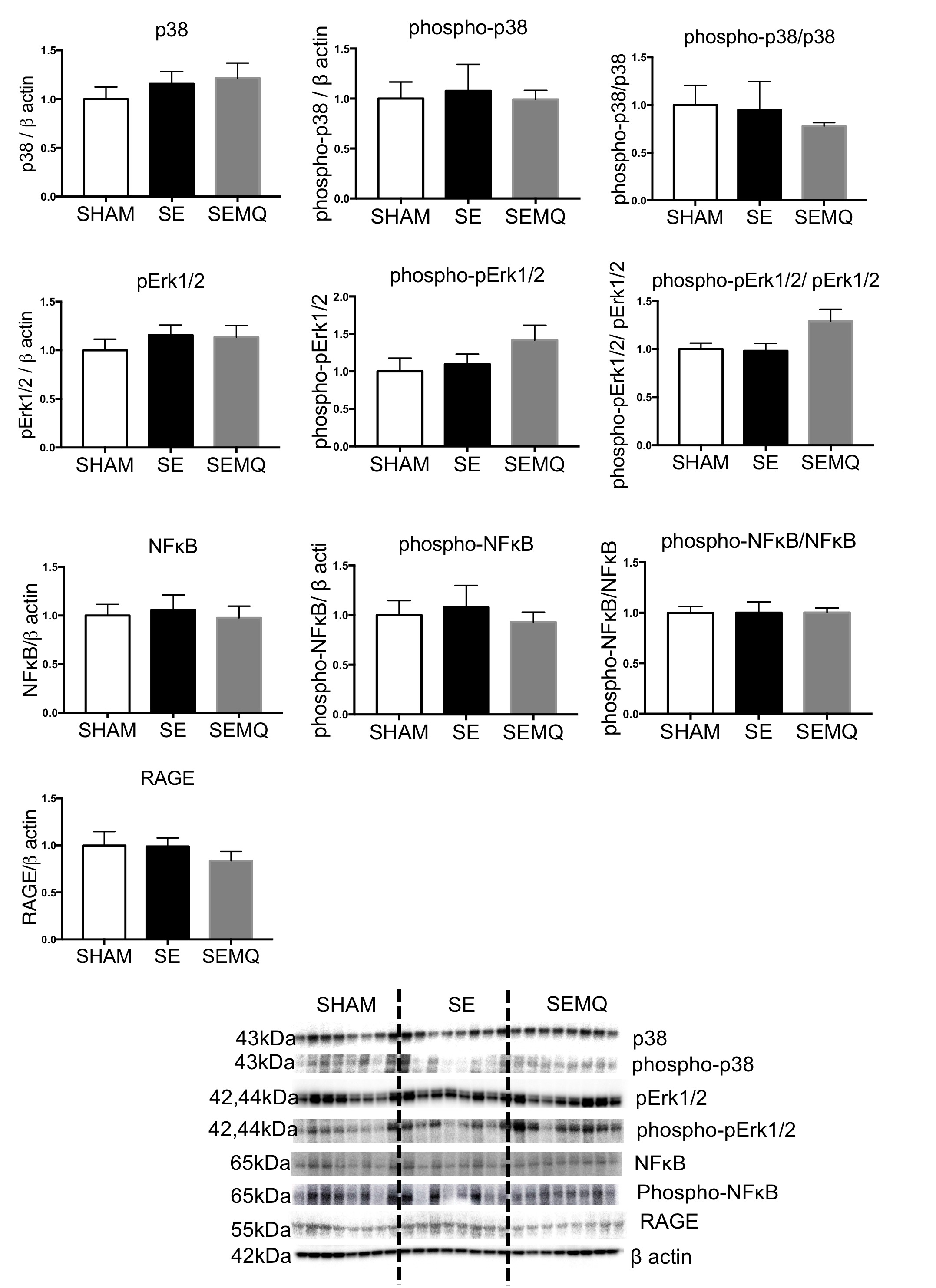


Supplementary figure 1. Renal protein level of markers on the RAGE pathway. Results are expressed as mean ± SE. Erk: extracellular signal-regulated kinase; JNK: c-JUN N-terminal kinase; p38MAPK: p38 Mitogen-activated protein kinase; NFκB: nuclear factor-κB; SE: cigarette smoke exposure; SEMQ: cigarette smoke exposure with MitoQ supplementation. Original gel images shown in supplementary figure 2(a-c).


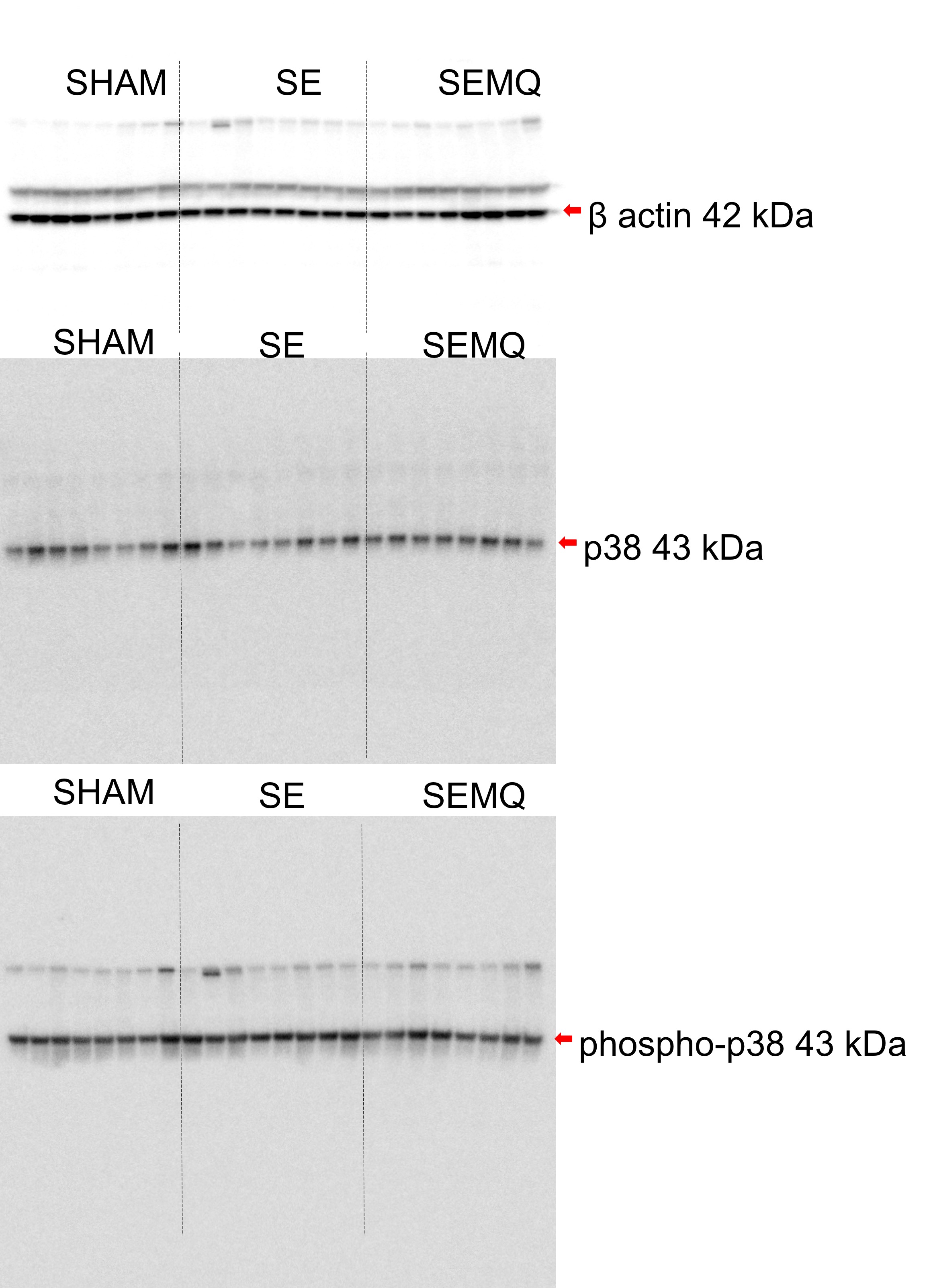


Supplementary figure 2a. Original blots for β actin, p38 and phospho-p38 in supplementary figure 1.


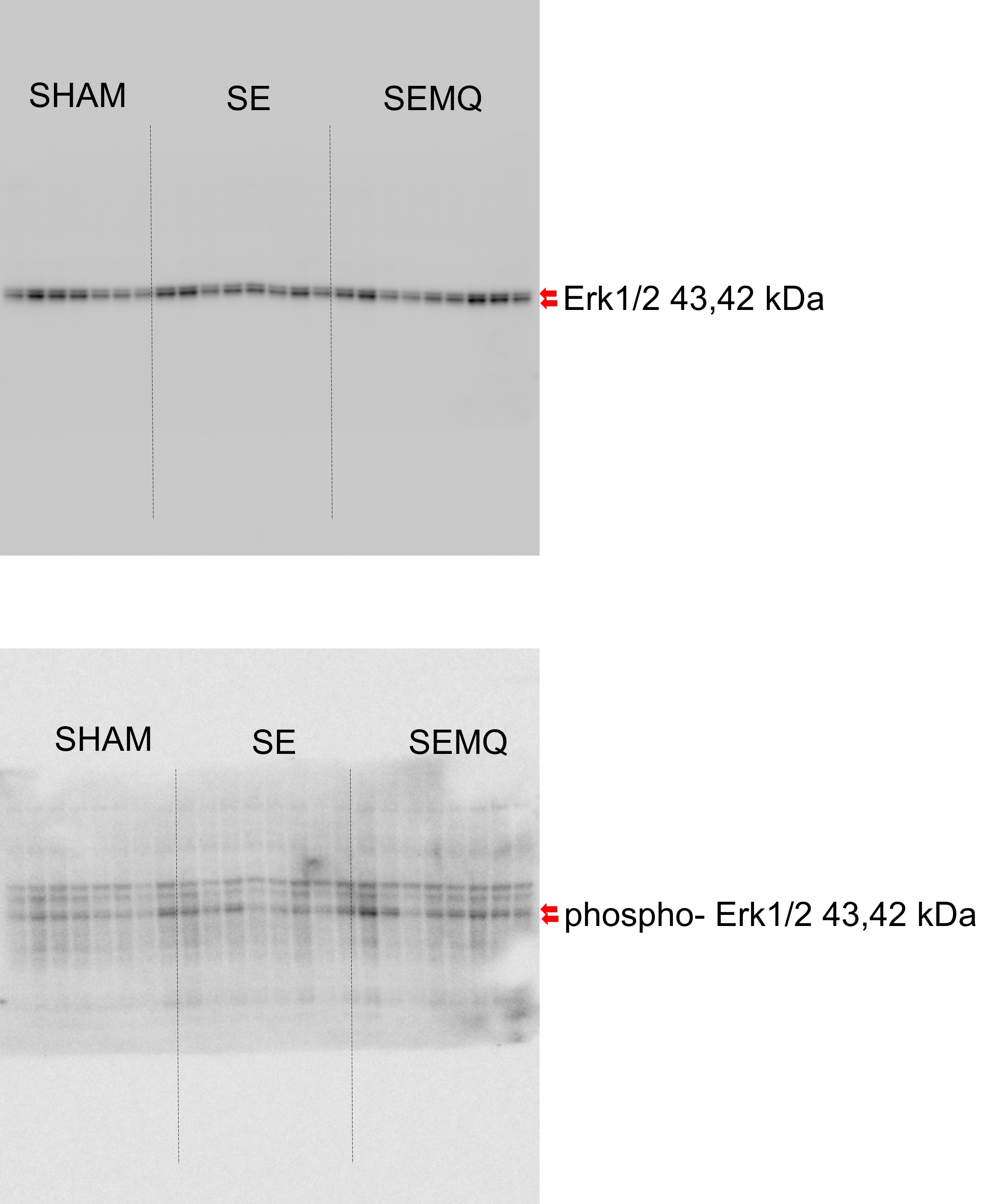


Supplementary figure 2b. Original blots for Erk1/2 and phospho-Erk1/2 in supplementary figure 1.


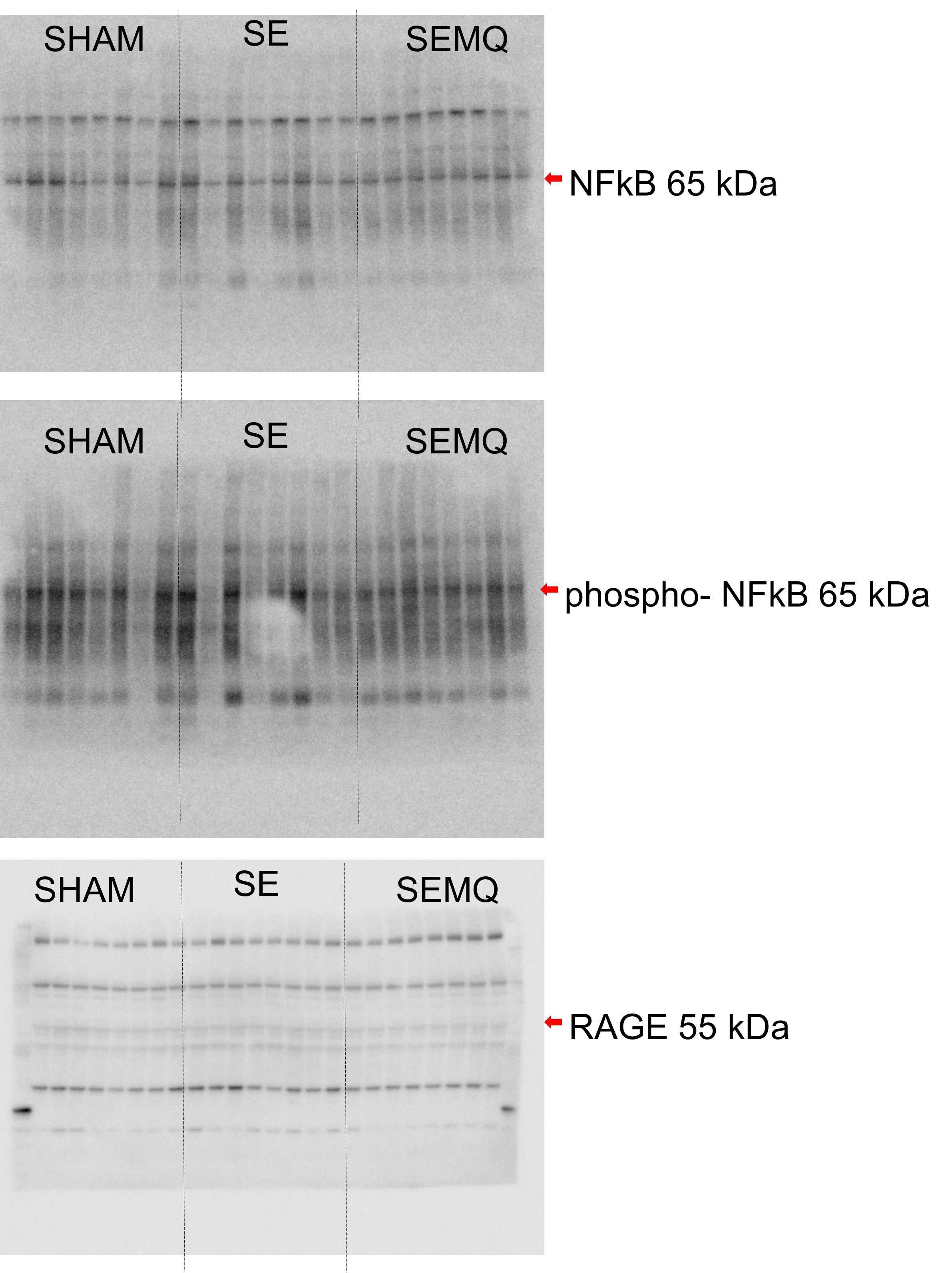


Supplementary figure 2c. Original blots for NFκB, phospho- NFκB and RAGE in supplementary figure 1.


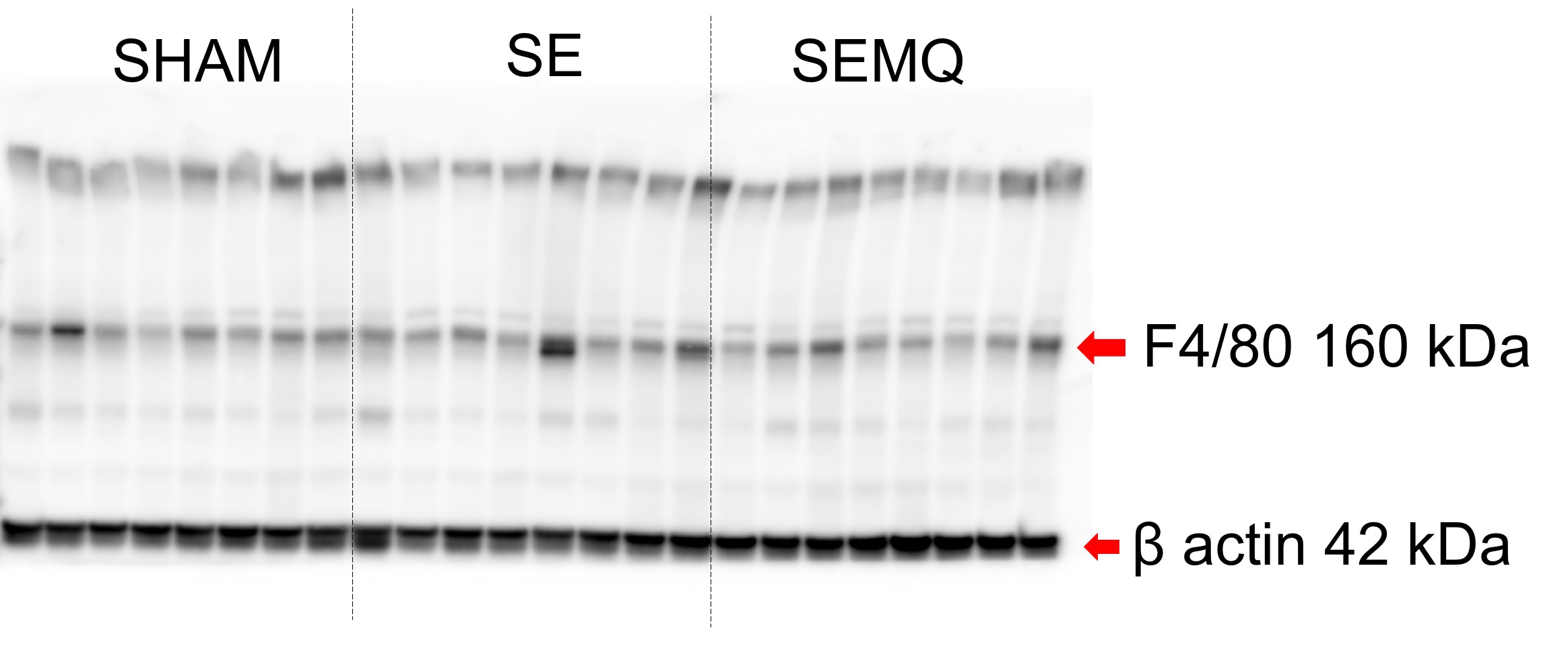


Supplementary figure 3. Original blots for F4/80 in figure 3.


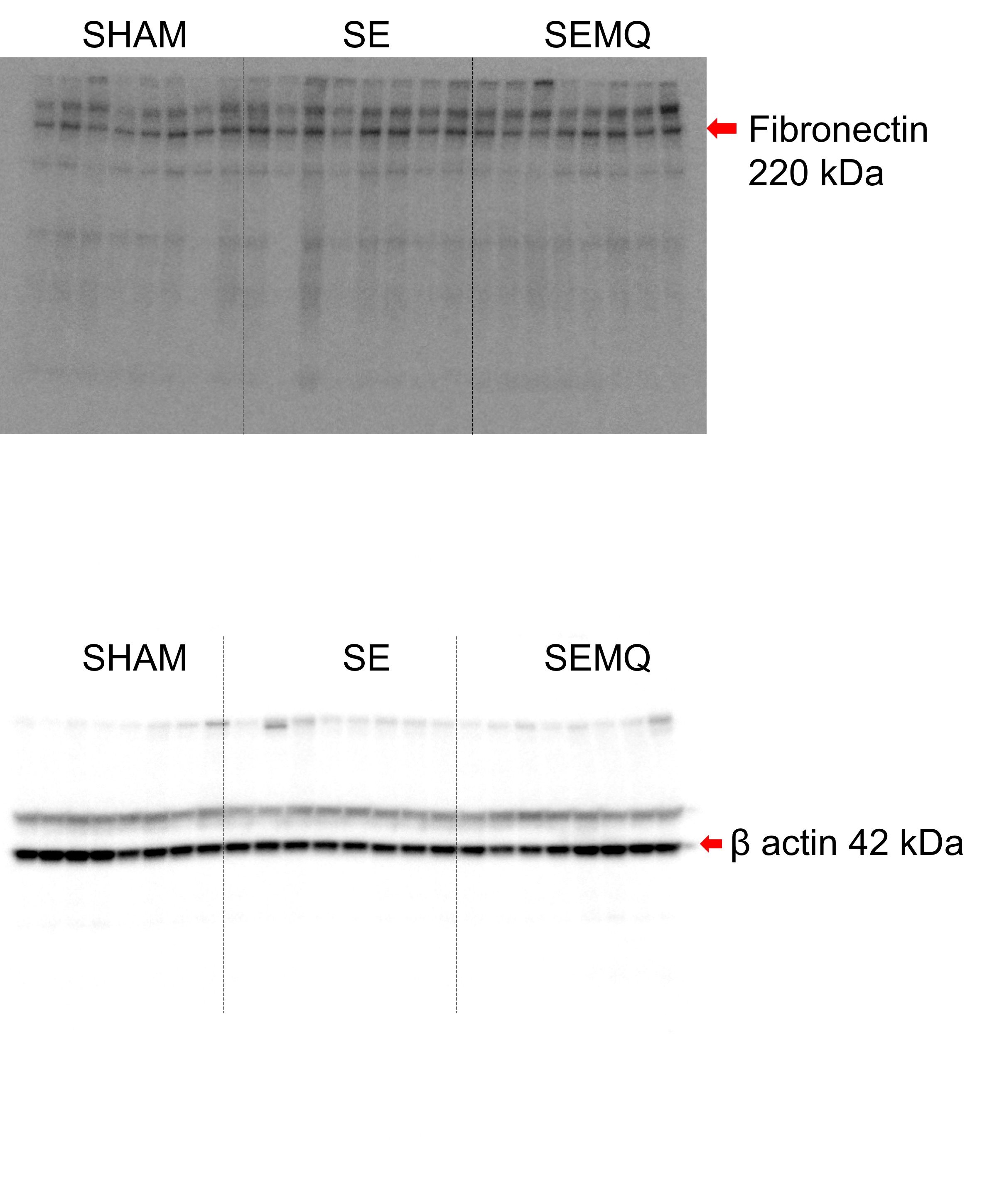


Supplementary figure 4a. Original blots for Figure 4a.


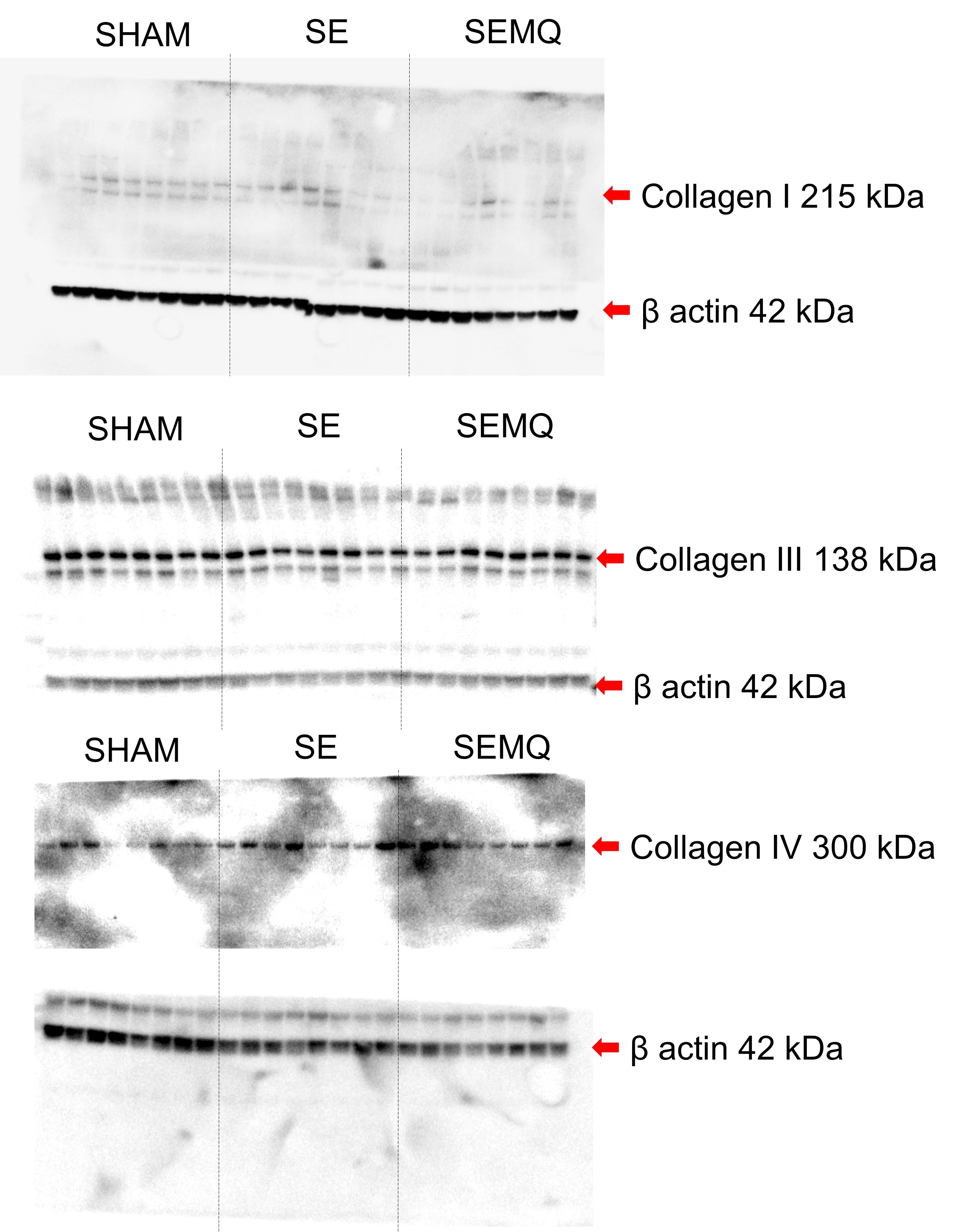
Supplementary figure 4b. Original blot for Figure 3b-d


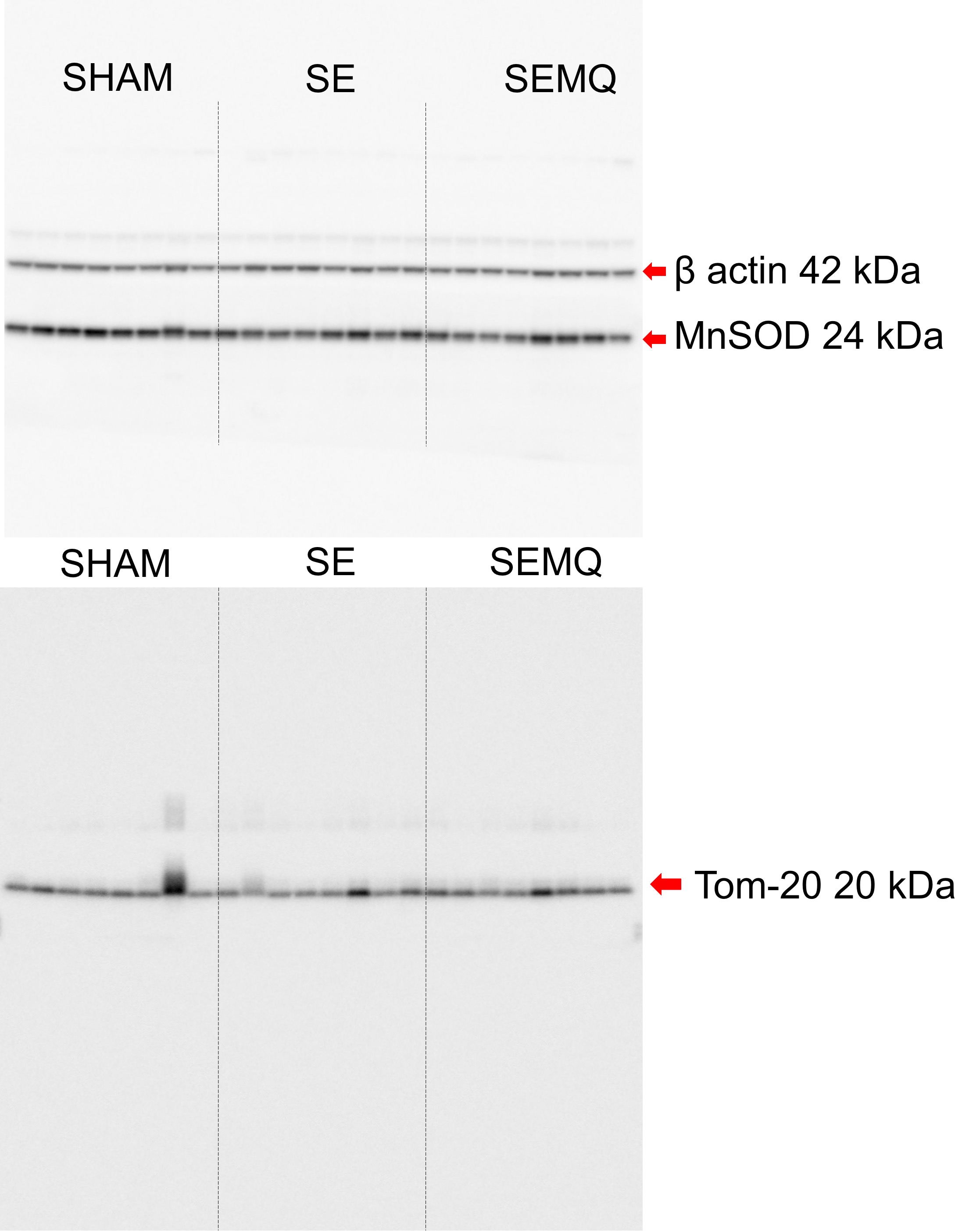


Supplementary figure 5. Original blots for Figure 5e and f.
